# Early Radiologic Diagnosis of Pulmonary Infection in Febrile Neutropenic Patients: A Comparison of Serial Chest Radiography and Single CT Chest

**DOI:** 10.1155/2021/8691363

**Published:** 2021-02-18

**Authors:** Wanaporn Burivong, Thanatorn Sricharoen, Apichart Thachang, Sunsiree Soodchuen, Panitpong Maroongroge, Vichit Leelasithorn

**Affiliations:** Department of Radiology, Faculty of Medicine, Srinakharinwirot University, Bangkok, Thailand

## Abstract

**Objective:**

The purpose of this study is to compare the early radiologic diagnosis of pulmonary infection between serial chest radiography (chest film) and single chest computed tomography (CT chest) in the first seven days of febrile neutropenia.

**Methods:**

This study included 78 patients with hematologic malignancies who developed 107 episodes of febrile neutropenia from January 2012 to October 2017 and had a chest film performed within the first seven days. Demographic and radiographic data were retrospectively reviewed. Three radiologists independently and blindly evaluated chest films and CT chests. The sensitivity, specificity, and correlation of chest film with absolute neutrophil count were carried out.

**Results:**

A total of 222 chest films were performed during this period and found thirty-nine episodes (36.4%) of radiographic active pulmonary infection. The diagnosis of clinical positive for pulmonary infection is 44.8% (48/107). Sensitivity, specificity, positive predictive value, and negative predictive value of serial chest film in the early radiologic diagnosis of pulmonary infection are 50%, 74%, 61%, and 64%, respectively. The false-positive rate was 14%, and the false-negative rate was 22%. For single CT chest examinations, twenty-six studies were assessed, and 42.3% was indicative of radiographic active pulmonary infection. Sensitivity, specificity, positive predictive value, and negative predictive value of CT chest in the early radiologic diagnosis of pulmonary infection are 91%, 40%, 53%, and 86%, respectively. The false-positive rate was 60%. The absolute neutrophil count was not useful for predicting radiographic active pulmonary infection.

**Conclusion:**

Serial chest film for early radiologic diagnosis of pulmonary infection within the first seven days of febrile neutropenia has lower sensitivity with higher specificity as compared to a single CT chest. Conversely, CT chest may not only have a higher sensitivity in determining early pulmonary infection but also has a higher rate of false-positives.

## 1. Introduction

Patients with febrile neutropenia are particularly at high risk of infection including bacteria, fungi, and viruses [1, 2]. About 85% of patients are found in underlying hematological malignancies who received chemotherapy or underwent a hematopoietic stem cell transplant. [1]. The type and strength of chemotherapy, as well as the duration and severity of neutropenia, are the main risk factors that promote infection in these patients [3]. Infection often occurs in the gastrointestinal and respiratory tract [1, 2]. Pulmonary infection develops in about 15%. Respiratory signs and symptoms in these patients are lower than usual. Therefore, chest film is included as a part of diagnostic workup. One of the common chest infections found in these patients is invasive pulmonary aspergillosis. The chest film in this infection can show subtle or maybe undetectable findings, making its early radiographic sensitivity and specificity rather low [4]. A prior study reveals 29% of negative chest films and 71% of nonspecific abnormal radiographs in neutropenic patients with early invasive pulmonary aspergillosis [3]. More obvious radiographic findings can be seen when the neutrophil count increases. Computed tomography (CT) in febrile neutropenic patients with suspected pulmonary infection shows higher sensitivity and specificity [5]. Specific CT appearance such as mass or nodule with halo sign, cavitary lesion, and air-crescent sign can be identified and gives more suggestion of invasive pulmonary aspergillosis [5, 6]. However, due to the higher cost and radiation dose level of CT over chest film, the proper timing for CT examination is essential. Investigation too early may have negative radiographic findings, while investigation too late may cause delay diagnosis and treatment.

The purpose of this study is to compare the early radiologic diagnosis of pulmonary infection in the first seven days of febrile neutropenia between serial chest films and single CT chest by determining their relative sensitivity and specificity.

## 2. Materials and Methods

### 2.1. Study Population

This retrospective study was carried out at the Department of Radiology, Faculty of Medicine, Srinakharinwirot University, after approval from the Institutional Ethics Committee. We included patients with the underlying hematologic disease who developed febrile neutropenia (neutrophil count <500 *μ*L and temperature >38.3°C) at HRH Princess Maha Chakri Sirindhorn Medical Center from January 1, 2012 to October 31, 2017. The inclusion criteria were patients who have chest film within the first seven days of febrile neutropenia. We excluded patients under age 15 and patients with unavailable clinical data or radiographic imaging.

### 2.2. Demographic Data Collection

The demographic data, age, sex, type of hematologic disease, chest symptom, and laboratory findings related to infection were recorded. Chest symptom was classified into cough, dyspnea, increased respiratory rate >20 min, decreased oxygen saturation <90%, and abnormal chest sounds such as rales, rhonchi, and decreased breath sound. Laboratory data related to infection in chest and outside chest were collected.

### 2.3. Radiographic Data

Three board-certified diagnostic radiologists independently and blindly reviewed chest film and CT chests within the first seven days of febrile neutropenia. Baseline chest film within three months before the episode of febrile neutropenia was also collected. Chest film opacity was classified into no opacity, new opacity, worsened opacity, stable opacity, and improvement. The chest films with no opacity, stable disease, and improvement were grouped as no radiological active pulmonary infection, while chest films with new lesions and progression were grouped as radiological active pulmonary infection. CT chest pattern was specified as pulmonary consolidation, centrilobular nodules/septal thickening, ground-glass opacity, nodule/mass, and atelectasis. Patients with CT chest findings of nodule/mass and atelectasis without other patterns were grouped as no radiological active pulmonary infection. Other CT chest findings are considered as radiological active pulmonary infection. In the cases where the radiologic interpretation was not unanimous, the agreement of two of the three radiologists was required for an interpretation to be considered final.

### 2.4. Statistical Analysis

The sensitivity, specificity, positive predictive value, and negative predictive value were determined by numbers of radiological active pulmonary infection from chest film and CT chest using the presence of either positive chest symptoms or positive mycobacterial study as a gold standard. Evaluation of the ability of absolute neutrophil count to differentiate positive chest films in febrile neutropenic patients was performed using area under the curve (AUC) of the receiver operating characteristic curve (ROC). Interobserver agreement of the radiographic findings on chest film and CT chest was calculated using Cohen's kappa (*K*) test.

## 3. Results

From January 2012 to October 2017, 78 patients with underlying hematologic malignancies had developed 107 episodes of febrile neutropenia. The mean age of patients was 47.9 years, with a range of 18–90 years. There were 47 male (60.3%) and 34 female (39.7%) patients. Of the 107 episodes, 41 (38.3%) had positive chest symptoms and 33 (30.8%) had laboratory data related to infection in the chest. The diagnosis of early pulmonary infection from the presence of chest symptoms or laboratory data or the combination thereof is 44.8% (48/107). Baseline characteristics of the patients, as well as chest symptoms and laboratory data, are described in Table 1.

A total of 222 chest films were performed during the first seven days of febrile neutropenia, 50% shows no opacity, while 15.8% had new lesions, 7.7% had progression, 19.8% was stable, and 7.2% found improvement on follow-up chest films. Thirty-nine episodes (36.4%) show radiographic active pulmonary infection, 14.0% was falsely positive, and 22.4% was falsely negative. Sensitivity, specificity, positive predictive value, and negative predictive value of serial chest films in the early radiologic diagnosis of pulmonary infection are 50%, 74%, 61%, and 64%, respectively.

**Figure 1:**
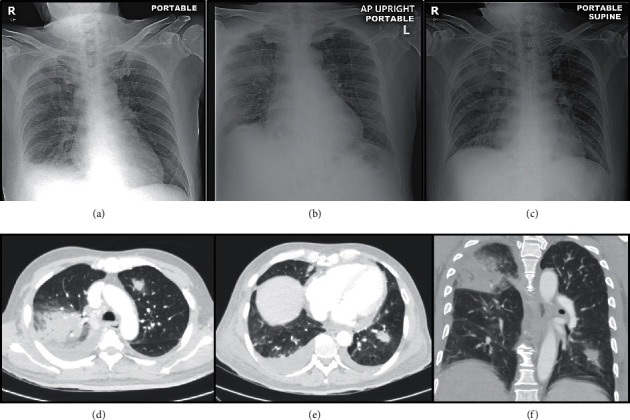
Images in a 58-year-old male patient with acute myeloid leukemia who had positive chest symptoms. (A) Baseline chest film shows right lower lung opacity. (B) Chest film on first day of febrile neutropenia (ANC = 0 *μ*L) shows new opacity at right upper lung and left retrocardiac region. (C) Chest film on the fifth day of febrile neutropenia (ANC = 624 *μ*L) shows stable opacities at right upper and left lower lungs. (D–F) Axial and coronal CT chest on third day of febrile neutropenia (ANC = 432 *μ*L) reveals pulmonary consolidation at right upper lobe and a nodular lesion at left lower lobe. Both lesions are surrounded by a halo of ground-glass attenuation, suggesting angioinvasive pulmonary aspergillosis. This case was evaluated as true-positive chest radiograph and CT chest.

**Figure 2:**
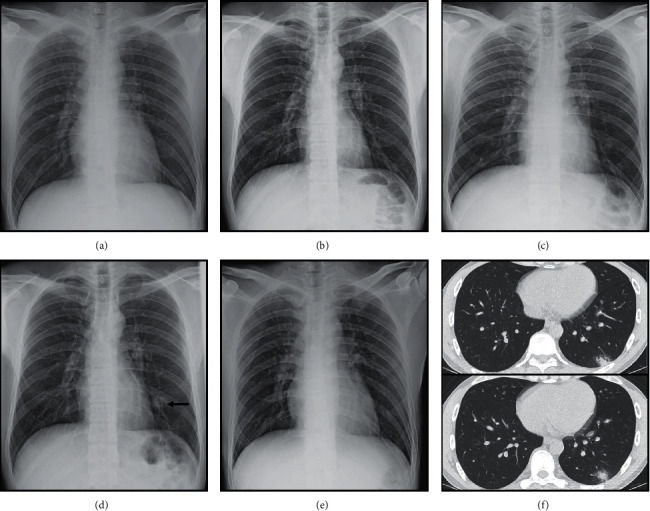
Images in a 37-year-old male patient with acute myeloid leukemia whose serum galactomannan test was positive. (a) Baseline chest film shows no opacity. (b) Chest film on first day of febrile neutropenia (ANC = 11 *μ*L) shows no opacity. (c) Chest film on third day of febrile neutropenia (ANC = 17 *μ*L) still shows no opacity. (d) Chest film on fourth day of febrile neutropenia (ANC = 0 *μ*L) shows new opacity at left lower lung (arrow). (e) Chest film on seventh day of febrile neutropenia (ANC = 2511 *μ*L) shows improvement of lower lung opacity. (f) Axial CT chest on the fourth day of febrile neutropenia reveals a nodular-like consolidation with surrounding ground-glass attenuation, representing a CT halo sign. This case was evaluated as true-positive chest radiograph and CT chest.

**Figure 3:**
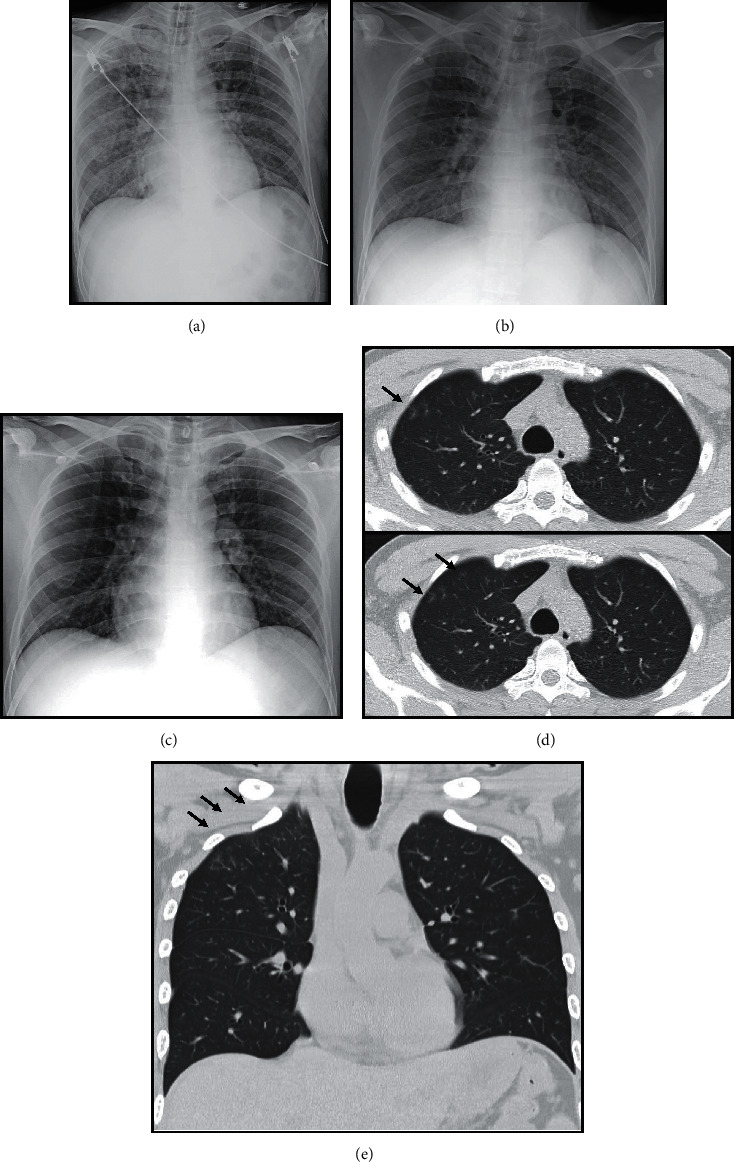
Images in a 35-year-old male with acute myeloid leukemia who had positive chest symptoms and serum galactomannan test. (A) Baseline chest film shows opacities at right upper and right lower lungs. (B) Chest film on second day of febrile neutropenia (ANC = 0 *μ*L) shows improvement of previous opacities with no new opacity. (C) Chest film on fifth day of febrile neutropenia (ANC = 0 *μ*L) shows no abnormal opacity. (D–E) Axial and coronal CT chest on seventh day of febrile neutropenia (ANC = 0 *μ*L) reveals subtle ground-glass attenuation at right upper lobe (arrows). This case was evaluated as false-negative chest radiograph and true-positive CT chest.

Not all patients underwent CT chest examinations. Twenty-six single CT chest studies were performed during this period, 19 of which (73%) were positive with the following rates: pulmonary consolidation 56.0%, ground-glass attenuation 40.0%, nodular or mass lesions 32.0%, atelectasis 20%, and centrilobular nodules 4%. Ten CT chests (38.4%) were indicative of radiographic active pulmonary infection, and 60% was falsely positive. Sensitivity, specificity, positive predictive value, and negative predictive value of single CT chest in the early radiologic diagnosis of pulmonary infection are 91%, 40%, 53%, and 86%, respectively. There are 36.8% (7/19) of positive CT chest, which found no radiographic active pulmonary infection from serial chest film. Examples of serial chest film and CT chest abnormalities are presented in Figures 1–3.

Details of consensus serial chest films and single CT chest compared to the clinical diagnosis of early pulmonary infection are summarized in Table 2.

**Figure 4:**
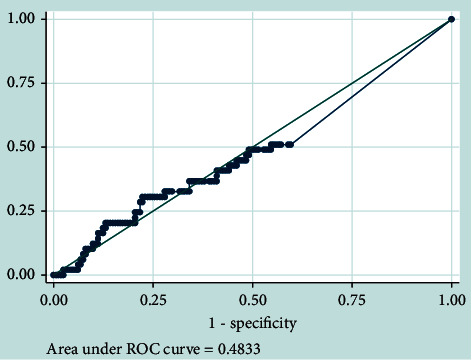
Receiver operating characteristic curve comparing the absolute neutrophil count with radiologic active pulmonary infection as predictors of pulmonary infection.

ROC analysis shows that the absolute neutrophil count was not useful for predicting radiographic active pulmonary infection (AOC 0.483) (Figure 4).

The overall agreement was substantial for the detection of baseline chest film lesions (*K* value 0.65). For identifying the presence of a lesion, interobserver agreement was higher when using a single CT chest as compared to serial chest films (*K* value 0.64 and 0.55). Details of the interobserver agreement are summarized in Table 3.

## 4. Discussion

Patients with underlying hematologic malignancies who received chemotherapy can develop febrile neutropenia. We found leukemia, lymphoma, and multiple myeloma as underlying hematologic diseases in febrile neutropenic patients, which are similar in order as Dimirel et al. [2]. When these patients develop febrile neutropenia, it leads to increased patient risk of infections, especially in the lungs. Compared to priors, our results show a higher rate of clinical pulmonary infection, likely due to patients who confirmed the diagnosis of infection outside the chest without any chest film and were therefore not included in our study population [7, 8]. The radiological active pulmonary infection compared to the clinical diagnosis of early infection in our study shows low sensitivity and moderate specificity. As compared to the study by Gerritsen et al. [9], our study reveals higher sensitivity but lower specificity. These different results may be due to different patient populations and timing of febrile episodes used for chest film interpretation. The false-negative findings are lower than in prior studies but still suggest that negative chest film does not exclude active radiologic pulmonary infection [8, 10]. The type of pulmonary infection can also affect false-negative rates in chest film. Invasive pulmonary mycoses are often not visible on chest film in the early neutropenic setting. CT chest can detect very early stage of invasive pulmonary mycoses and improve the detection rate of pulmonary infiltration [9]. Zaleska-Dorobisz et al. reported a higher sensitivity using low-dose CT chest than single chest film for detection and characterization of pulmonary abnormalities in pediatric patients with neutropenic fever. However, the higher radiation dose for CT is a concern [10]. Our single CT chest in the first seven days of febrile neutropenia shows higher sensitivity and negative predictive value than serial chest films for the detection of early pulmonary infection. Similar results were found in malignant hematological patients who received chemotherapy or underwent hematopoietic stem cell transplant (HSCT) with chest CT and chest film performed on the first day of febrile neutropenia [9]. Other studies show an increased detection rate from chest CT compared to chest film but differ in some points from our study [11–13]. However, there is a lower specificity for early pulmonary infection detection from a single CT chest than serial chest films and a high false-positive rate from the single CT chest in our study. Serial chest films can show changes or stability of the pulmonary opacities, which may improve radiologists' confidence in determining active or not active radiological infection. Conversely, a single CT chest with the presence of specific CT patterns or specific radiologic stigmata may also increase the differentiation between infectious and noninfectious pulmonary disease, as well [12]. A study by Cornetto et al. shows treatment benefit for both normal and abnormal early CT chest after HSCT [14]. However, our study did not examine this point in detail. Another detail that could cause lower than expected specificity is our categorization of nodule/mass as no radiologic active pulmonary infection for the purposes of our study, while in fact, some nodules/mass lesions are infectious processes. Therefore, a single CT chest should be helpful for neutropenic patients with fever of unknown origin.

About the relationship between absolute neutrophil count and chest film suggesting active pulmonary infection by using the ROC curve, we found the absolute neutrophil count is not a significant predictive value for radiologic active pulmonary infection. This result is defined by the low neutrophil count, which limits the inflammatory response in the chest and leads to undetectable pulmonary infiltration evidenced by radiography [7]. Deborah et al. revealed that the utility of chest film in diagnosing infection was low in the absence of respiratory symptoms. The use of serial chest film to evaluate febrile neutropenic patients with respiratory signs or symptoms can be applied [11, 13].

The variability of radiology interpretation assessed by analysis of interobserver agreement shows a similar value as informed by Gerritens et al. for chest film interpretation. The agreement for CT interpretation from our study was slightly higher [9].

There were a few limitations to this study. First, not all first seven days of febrile neutropenia have chest films and not all episodes have a CT chest. Most of the CT chests were requested if there was the presence of clinical or chest film suspicious of pulmonary abnormality. Second, our patient population does not include those with clinical diagnoses confirmed of infection outside the chest who have no chest film. Third, laboratory data related to respiratory tract infection were not reported in all patients with suspected pulmonary infection due to the retrospective nature of the study.

In conclusion, serial chest film for early radiologic diagnosis of pulmonary infection within the first seven days of febrile neutropenia has lower sensitivity with higher specificity as compared to a single CT chest. However, there is a higher false-positive rate for CT chest in determining early pulmonary infection. The number of absolute neutrophil count cannot predict the presence of radiologic active pulmonary infection from chest film. As a result, we could apply the use of serial chest films for early febrile neutropenia workup in patients with respiratory signs and symptoms, while CT chest will be more suitable when neutropenic patients have fever of unknown origin. Another advantage of serial chest film over CT chest is for the pediatric population, for which radiation dose is concern, and in low immune patients who need isolation. [15].

## Figures and Tables

**Table 1:** Baseline characteristics of the patients, including details of chest symptoms and laboratory data related to chest infection.

Baseline characteristic	Features	Number (%)
Gender	Male/female	47/31 (60.3/39.7)
Age (years)	Median	47.9 (range 18–90)
Hematologic malignancies	Leukemia	43 (55.1)
	Lymphoma	31 (39.7)
	Multiple myeloma	4 (5.1)

Chest symptoms	Positive/negative	41/66 (38.2/61.7)
	Shortness of breath	21 (51.2)
	Cough	19 (46.3)
	Tachypnea	15 (36.6)
	Lung signs	7 (17.1)
	Decreased breath sound	4 (9.8)
	Hypoxia	2 (4.9)
	Hemoptysis	1 (2.4)

Laboratory data related to chest infection	Positive/negative	33/74 (30.8/69.2)
	Microbial culture	18 (54.5)
	Serum galactomannan	18 (54.5)
	Influenza test	2 (6.0)

**Table 2:** Summary of consensus chest films and CT chest compared to the clinical diagnosis of early pulmonary infection.

Early pulmonary infection		Clinical positive (*n* = 48)	Clinical negative (*n* = 59)	
Chest film	Radiological active (*n* = 39)	24	15	PPV 61%
	No radiological active (*n* = 68)	24	44	NPV 64%
Early pulmonary infection		Clinical positive (*n* = 11)	Clinical negative (*n* = 15)	
CT chest	Radiological active (*n* = 19)	10	9	PPV 45.8%
	No radiological active (*n* = 2)	1	6	NPV 100%

PPV, positive predictive value; NPV, negative predictive value.

**Table 3:** Interobserver agreement in chest film and CT chest interpretation.

Radiographic interpretation	Observer			
	1 and 2	2 and 3	3 and 1	All
Baseline chest film	0.60	0.66	0.71	0.65
Presence of chest film active pulmonary infection	0.56	0.56	0.55	0.55
Presence of CT chest active pulmonary infection	0.61	0.69	0.62	0.64

## Data Availability

The data used to support the findings of this study are available from the corresponding author upon request.
